# Pelvic Exenteration for Advanced and Recurrent Gynecologic Malignancies: Surgical Morbidity and Survival Outcomes From a Tertiary Cancer Center

**DOI:** 10.7759/cureus.113979

**Published:** 2026-08-05

**Authors:** Rachana Palakkal, Adarsh Dharmarajan, Ashitha R Gangadharan, Nila S Pradeep

**Affiliations:** 1 Gynecologic Oncology, Malabar Cancer Centre, Thalassery, IND

**Keywords:** disease free survival, gynecologic malignancies, overall survival, pelvic exenteration, post operative complications

## Abstract

Introduction

Pelvic exenteration (PE) involves en-bloc removal of central pelvic organs and is indicated for patients with locally advanced or recurrent cervical, endometrial, ovarian, vaginal, and vulvar cancers. Our study aimed to analyze the surgical morbidity and survival in patients who underwent pelvic exenteration surgery for gynecological malignancies.

Methods

Sixty patients underwent pelvic exenteration for gynecological malignancy at our centre from January 2013 to December 2020. We included all patients who underwent a pelvic exenteration procedure for locally advanced or recurrent cervical, ovarian, endometrial, vaginal and vulvar cancers with both curative and palliative intent. The primary objective was to analyze the perioperative complications, pattern and severity of perioperative morbidity, and the requirement for reconstruction procedures. The secondary objective was to analyze the disease-free survival (DFS) and overall survival (OS) associated with this radical procedure

Results

Among 60 patients who underwent the exenteration procedure, total, anterior, and posterior exenteration was performed in two (3.33%), two (3.33%), and 56 (93.33%) patients, respectively. Intraoperative complications occurred in 13 cases. Median follow-up period was 50 months. The OS rates were 87.7% at one year, 78.5% at two years, 58% at three years, and 36.6% at four years and DFS showed a similar declining trend over time, with 75.1% of patients remaining disease-free at one year, 50.0% at two years, 28.0% at three years, and 25.7% at four years.

Conclusion

In summary, pelvic exenteration is a definite option in indicated cases of advanced gynecological malignancies in the background of advanced surgical techniques, modern intensive care facilities, and surgical expertise. With respect to survival benefits, exenteration procedures are associated with an acceptable rate of major complications

## Introduction

Pelvic exenteration (PE) was initially described by Brunschwig in 1948 as a palliative procedure for managing symptoms associated with locally advanced gynecologic cancers, but it has evolved into a curative surgical option [1]. This shift is greatly due to improvements in medical technologies and stricter criteria for selecting appropriate candidates. The procedure involves en-bloc removal of central pelvic organs and is indicated for patients with locally advanced or recurrent cervical, endometrial, ovarian, vaginal, and vulvar cancers [2,3]. Pelvic exenteration is categorized into anterior, posterior, and total [4].

Pelvic exenteration is an extensive surgical procedure designed to achieve complete tumor clearance through the resection of all involved pelvic organs. Depending on the extent of disease, the surgery may include resection of both gastrointestinal and genitourinary structures, such as the distal sigmoid colon, rectum and anus, bladder and urethra, as well as the uterus, ovaries, and vagina. Earlier PE was considered unsuitable for patients with lateral pelvic sidewall involvement; Höckel et al. proposed laterally extended endopelvic resection (LEER) for complete resection in patients with lateral pelvic recurrence or locally advanced gynecologic malignancies [5,6]. This technique includes resection of the medial acetabulum, obturator membrane, sacrospinous ligament, components of the sacral plexus, and the piriformis muscle, along with the entire internal iliac vascular compartment [7,8].

Complication rates, both early and late, vary from 21.3% to 94.4% [2,9] depending on the extent and complexity of the surgery. Infection was the most common complication, followed by wound dehiscence, urinary complications, hemorrhage, ileus, fistulae, anastomotic leaks, and complications related to reconstruction procedures [2,3]. Perioperative mortality ranges between 0 and 6% [2,10]. Due to heterogeneous study populations, survival rates differ between studies. In similar studies, five-year overall survival (OS) ranges from 27.4 to 55% and progression-free survival (PFS) from 34.4 to 36.0% [10-13]. To date, literature on risk factors influencing morbidity and survival in patients undergoing pelvic exenteration remains sparse.

The primary objective of this study was to determine the surgical outcome and perioperative complications of pelvic exenteration surgeries for gynecological malignancy. The secondary objective was to analyze the disease-free survival (DFS) and OS associated with this radical procedure.

## Materials and methods

This was a retrospective study done in a tertiary care cancer centre in South India. The study was approved by the institutional review board vide no; 1616/IRB-SRC/13/MCC/28-10-23/6. Sixty patients underwent pelvic exenteration for gynecological malignancy at our centre from January 2013 to December 2020. We included all patients who underwent a pelvic exenteration procedure for locally advanced or recurrent cervical, ovarian, endometrial, vaginal, and vulvar cancers with both curative and palliative intent.

Patient demographics, clinical and radiological findings, stage, modality of treatment, lymph node status, postoperative events, and follow-up details are recorded in case files. All patients were initially evaluated by a gynecologic oncologist, and the treatment plan was made after a multispeciality board comprising a medical oncologist, radiation oncologist, gynecologic oncologist, oncopathologist, and radiologist.

OS was defined as the interval between surgery and death from any cause or last day of follow-up. DFS was defined as the interval between the surgical procedure and the date of progression or recurrence, last day of follow-up, or death from any cause. Pelvic exenteration is categorized into anterior, posterior, and total. Anterior pelvic exenteration is the removal of the bladder along with the uterus; modified posterior exenteration is the removal of the rectum along with the uterus, and total involves removal of the bladder, uterus, and rectum.

Clinical details of patients who underwent a pelvic exenteration procedure from January 2013 to December 2020 at the division of Gynecologic Oncology at Malabar Cancer Centre, Thalassery, Kerala were retrospectively collected. We included all patients who underwent pelvic exenteration procedure for locally advanced or recurrent cervical, ovarian, endometrial, vaginal and vulvar carcinoma with both palliative and curative intent. The institutional policy was to offer pelvic exenteration for patients with rectal involvement. In our study, R0 resection was strictly defined as complete tumor resection with no microscopic residual disease, i.e., all surgical margins were free of tumor on histopathological examination. Surgical management was undertaken with curative intent in all eligible patients, following strict adherence to en bloc resection principles to ensure complete tumor removal. Every effort was made intraoperatively to achieve negative resection margins, with comprehensive resection of involved organs and adjacent structures as indicated, in accordance with standard oncologic surgical practices.

Patient demographics, including body mass index (BMI) and comorbidities, were collected from both digital and archived medical records. We also collected clinical details, such as characteristics of the disease, including site of tumour and indication for pelvic exenteration, histopathological features, prior treatments, neoadjuvant treatment (chemotherapy or radiation therapy), and peri- and postoperative events. Pelvic exenteration was classified as anterior, posterior, or total exenteration, according to Magrina et al. [4]. Postoperative complications were classified using the Clavien-Dindo classification system for surgical complications [14].

Surgical procedures were categorized based on the extent of pelvic visceral resection. Classic pelvic exenteration (anterior or total) involves complete visceral extirpation and is typically reserved for central pelvic recurrences (e.g., cervical cancer). In our series, posterior exenteration (93.33%, n = 56) refers to a modified posterior pelvic exenteration (en bloc retroperitoneal resection of the rectosigmoid colon, uterus, and adnexa). In the context of advanced ovarian cancer (86.7% of cases), these procedures were performed as part of an ultra-radical cytoreductive surgery strategy with the primary objective of achieving complete macroscopic tumor clearance (R0 status).

If postoperative morbidity was reported as Grade IIIa or higher, the patient was classified as having a major postoperative complication. Any complications that occurred within six weeks of surgery were classified as early onset and as late onset if they occurred after six weeks. Perioperative death was defined as mortality that occurred within 30 days of surgery. Follow-up time was calculated from the date of surgery until the last date of follow-up. All patients were followed up three-monthly for the first two years, six-monthly till five years, and annually thereafter.

Statistics

Continuous variables were expressed as mean±standard deviation while categorical variables were expressed as frequencies and percentages. Survival analysis (both PFS and OS) was done using the Kaplan-Meier method. Statistical analysis was done using SPSS version 25.0.0 (IBM Corp., Armonk, USA). A p < 0.05 was statistically significant.

## Results

Characteristics of patient and disease

Between January 2013 and December 2020, 60 patients underwent pelvic exenteration for gynecological malignancy, and those who met the inclusion criteria were considered for our study and are displayed in Table 1. Mean age was 54 years, and average BMI was between 19.1 and 24.9. Among 60 patients, 14 (23.33%) were of the premenopausal group. It was observed that all patients who underwent the procedure had an Eastern Cooperative Oncology Group (ECOG) performance status score of one. In our study, 37 (61.66%) patients had some medical comorbidities requiring treatment, and the most common was diabetes mellitus, 26 (43.33%), followed by systemic hypertension, 22 (36.66%). The primary indication for pelvic exenteration was ovarian cancer, 52 (86.7%), followed by four (6.7%) endometrial carcinoma, three (5.0%) cervical, and one (1.7%) vaginal cancer. Among ovarian cancers, two (3.33%) were of International Federation of Gynecology and Obstetrics (FIGO) stage IC, 38 (63.3%) patients were stage IIIC, nine patients (15.0%) were of Stage IVA, and three (5.0% ) were stage IVB. In total, three patients (5%), two (3.4%) cases of ovarian cancer and one (1.7%)case of cervical cancer, underwent pelvic exenteration for recurrent disease. Among endometrial cancer cases, three (5%) were of FIGO stage IVA and one (1.7%) case was of stage IIIC. All were performed with curative intent. Patients with comorbidities were assessed using the Charlson comorbidity index, and 49 (82%) were scored less than five.

**Table 1:** Showing patient characteristics of pelvic exentration for gynecologic malignancies at a tertiary care cancer centre (n = 60) ECOG: Eastern Cooperative Oncology Group.

Characteristic	Category / Description	n (%)
Age (years)	Range; Median	54 (51–60)
Body Mass Index (kg/m²)	Range	19.1–24.9
ECOG Performance Status	Score 1	60 (100)
Previous Surgeries	Hysterectomy	6 (10.0)
	Splenectomy	1 (1.7)
Type of Pelvic Exenteration	Modified posterior pelvic exenteration	56 (93.3)
	Anterior pelvic exenteration	2 (3.3)
	Total pelvic exenteration	2 (3.3)
Operating Time	< 4 hours	6 (10.0)
	4–6 hours	22 (36.7)
	6–8 hours	27 (45.0)
	8–10 hours	5 (8.3)
Resection Margin Status	R0 resection	60 (100)
Reconstruction Procedures	Ileostomy	2 (3.3)
	Colostomy	8 (13.3)
	Ileal conduit	4 (6.7)
	Ileal conduit + colostomy	2 (3.3)
Associated Procedures	Lymph node dissection	56 (93.3)
	Total peritonectomy	8 (13.3)
	Pelvic peritonectomy	4 (6.7)
	Splenectomy	5 (8.3)
	Cholecystectomy	3 (5.0)
	Nephrectomy	1 (1.7)
	Liver surface deposit excision	4 (6.7)
	Axillary dissection	1 (1.7)

Preoperative treatment

Most patients, 46 (76.67%), had received neoadjuvant chemotherapy for ovarian cancer with paclitaxel and carboplatin. Three patients (5%) with recurrent cervical cancer received prior radiotherapy in the pelvic area. Seven patients (11.66%) had already undergone hysterectomy previously.

Surgery details

Among 60 (100%) patients who underwent the exenteration procedure, total, anterior, and posterior exenteration was performed in two (3.33%), two (3.33%), and 56 (93.33%) patients, respectively. As for the surgical procedure, all (100%) underwent a laparotomy procedure. The median operating time was 360 (range 150-540) minutes. R0 resection status was attained in all the cases (100%). Pelvic and para-aortic lymph node dissection was done in 56 (93.33%) patients and bilateral inguinofemoral lymph node dissection in one (1.7%) patient. Other procedures include eight (13.33%) total peritonectomies, four (6.66%) pelvic peritonectomies, three (5%) cholecystectomies, four (6.66%) liver surface deposit excisions, one (1.7%) nephrectomy, and one (1.7%) axillary dissection.

Intraoperative complications occurred in 13 (21.66%) cases, including hypotension in five (8.33%) cases, atrial fibrillation in one (1.7%) case, hypotension with atrial fibrillation in three (5%) cases, two (3.4%) cases of ureter injury, two (3.4%) cases of bladder injury, and one (1.7) case had inferior vena caval injury. Median blood loss estimated was 900ml (range 300-2500ml), and 35 (58.33%) patients required blood transfusion. Median hospital stay was nine days. Relaparotomy was done in five (8.33%) cases within 30 days; three (5%) cases had anastomotic leak, one (1.7%) case of ureterovaginal fistula, and one (1.7%) case of rectovaginal fistula. The most common postoperative complication was wound infection, six (10%). Postoperative complications were graded according to the Clavien-Dindo classification. Overall, 48 patients (80%) experienced minor complications (Grade I-II). Grade III complications, requiring surgical, endoscopic, or radiological intervention, were observed in six (10%) patients. Five (8.3%) patients developed Grade IV complications, representing life-threatening events requiring intensive care management. There was one (1.7%) postoperative mortality on postoperative day three due to sepsis (Grade V). None of the factors such as age group (0.847), BMI (p = 0.459), stage (p = 0.182), and performance status (p = 0.864) influenced postoperative complications. Among patients who underwent modified posterior pelvic exenteration, diversion stoma was done in 10(16.66%) patients, two (3.4%) patients underwent ileostomy, and eight (13.33%) underwent colostomy.

In postoperative pathology reports, among 52 (86.7%) ovarian cancers, 46 (76.66%) had high-grade serous carcinoma, three (5%) had mucinous carcinoma, two (3.4%) had clear cell carcinoma, and one (1.7%) case of dysgerminoma. Among four (6.66%) endometrial cancers, three (5%) were serous carcinoma and one (1.7%) case of clear cell carcinoma. In cervical cancers, two (3.4%) had squamous cell carcinoma, one (1.7%) had adenocarcinoma, and one (1.7%) was clear cell carcinoma of the vagina.

Survival

Median follow-up period was 50 months. R0 was achieved in all cases. A total of 52 (86.66%) cases of ovarian malignancies were treated, and 28 (46.66%) developed recurrence. Among endometrial cancer cases, one (1.7%) patient developed recurrence with peritoneal metastasis, and three (5%) patients died of other causes. All three (5%) exenteration procedures done for cervical cancer were for local recurrence. One case (1.7%) of carcinoma vagina expired due to myocardial infarction after 21 months of treatment.

**Figure 1:**
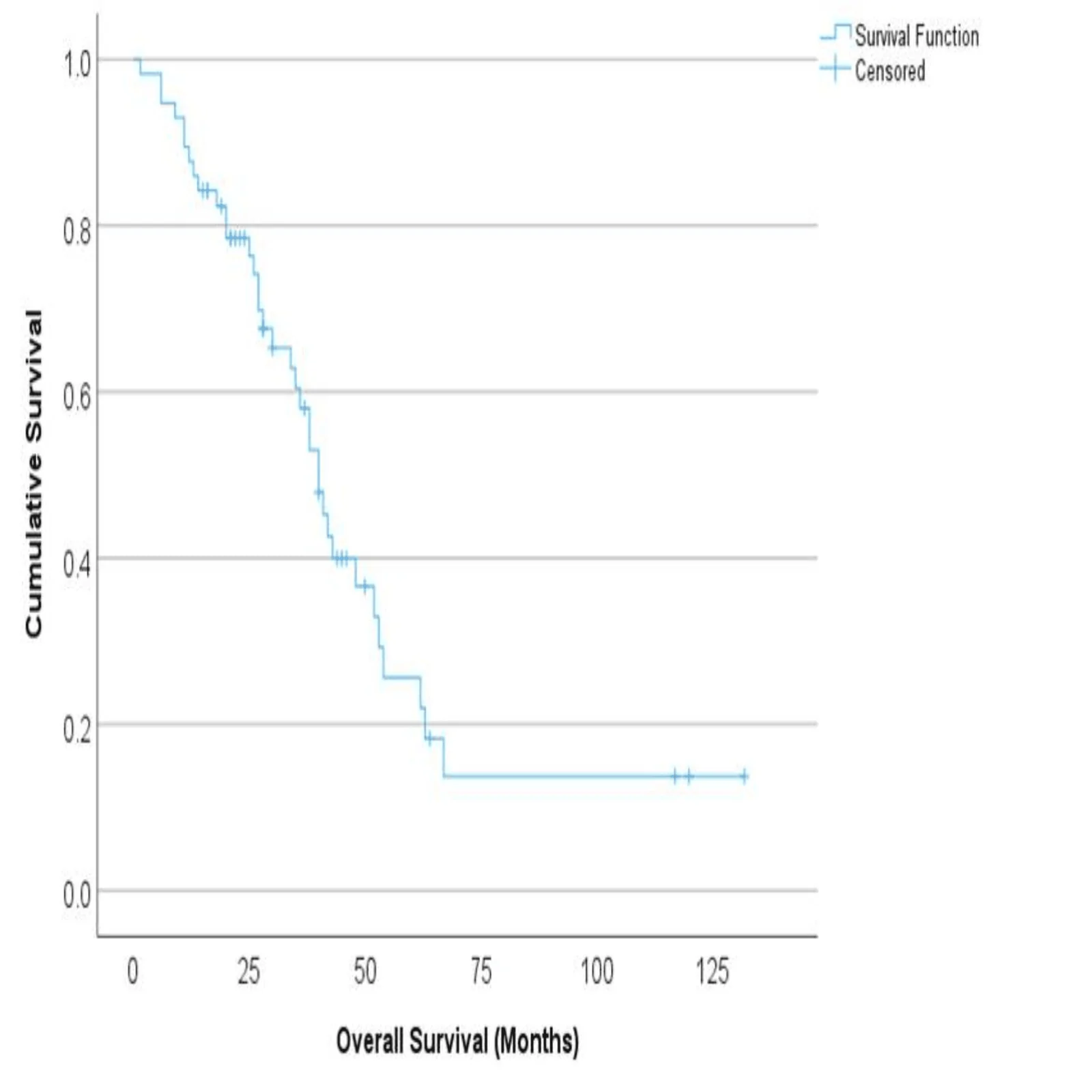
Kaplan meier curve showing overall survival of pelvic exentration for gynecologic malignancies at a tertiary cancer centre

**Figure 2:**
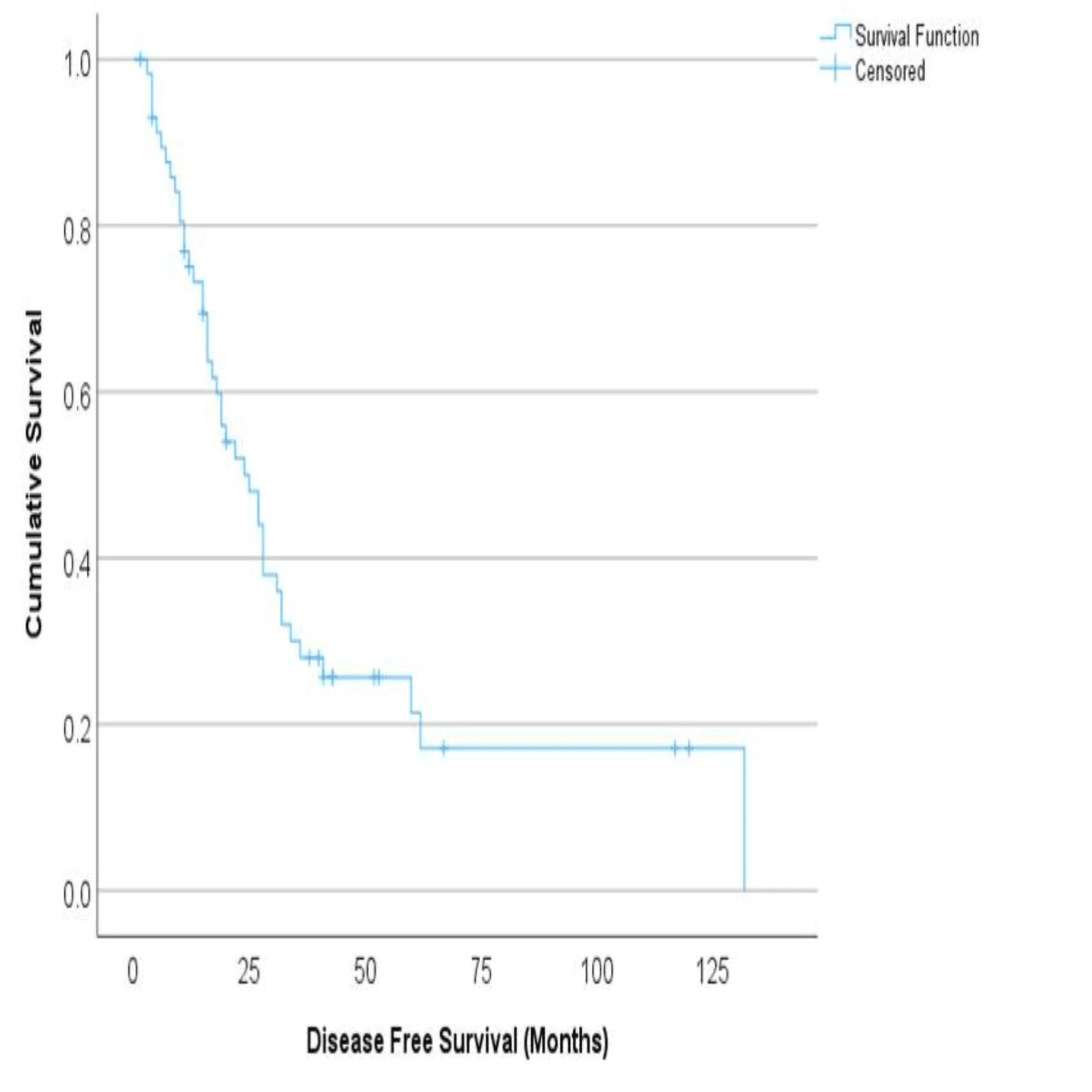
Kaplan meier curve showing disease free survival (DFS) of pelvic exentration for gynecologic malignancies at a tertiary cancer centre

The Kaplan-Meier analysis of patients undergoing pelvic exenteration for gynecologic malignancies at our tertiary cancer center demonstrated encouraging survival outcomes. The OS rates were 87.7% at one year, 78.5% at two years, 58% at three years, and 36.6% at four years (Figure 1). DFS showed a similar declining trend over time, with 75.1% of patients remaining disease-free at one year, 50.0% at two years, 28.0% at three years, and 25.7% at four years (Figure 2). Median OS and DFS were 40.0 months (95 CI: 34.2-45.7) and 24.0 months (CI: 14.9-33.0), respectively. These results highlight that while early post-operative survival is favorable, a gradual decline is observed over subsequent years, reflecting the risk of recurrence or disease progression in this high-risk population. None of the factors such as age, BMI, stage, and performance status influenced DFS or OS.

## Discussion

It is essential to distinguish modified posterior pelvic exenteration performed during ultra-radical cytoreduction for ovarian cancer from classic salvage pelvic exenteration performed for recurrent cervical or vaginal malignancies. In classical pelvic exenterations, the surgical goal is central pelvic clearance in a previously irradiated field, frequently requiring permanent urinary or fecal diversions. In contrast, 93.33% of patients in our cohort underwent posterior exenterative procedures primarily as an en bloc surgical approach to achieve complete cytoreduction (R0 resection in 100% of cases) in advanced ovarian cancer.

In this study, we evaluated the perioperative complications and survival in 60 patients who underwent exenteration surgery for gynecological malignancy. Thirteen (21.7%) patients had intraoperative complications. Postoperative complications occurred in 12 (20%) patients, with six (10%) cases categorized as major. 

Compared with the existing literature, the postoperative complication profile in our cohort appears lower than that reported in several major studies. In a study by Moolenaar et al., involving 90 patients, 83 patients (92.2%) experienced at least one postoperative complication [15]. Minor complications (Clavien-Dindo grade I-II) occurred in 31.3%, while major complications (grade ≥ IIIa) occurred in 61.1% of cases. These rates are considerably higher than those observed in our study, where most complications were minor, and only one patient experienced Grade 5 morbidity due to neutropenic sepsis.

Similarly, in another study by Glane et al. of 47 patients, 40.4% developed major complications [16]. The most frequent major complications included urinary tract obstruction requiring percutaneous nephrostomy (8.5%, n = 4) and wound healing problems (8.5%, n = 4). Their early mortality rate (within 30 days) was 4.3%, with two deaths-one due to E. coli sepsis and another due to hemorrhagic complications. In contrast, our study recorded only one (1.7%) postoperative death, attributable to neutropenic sepsis.

Overall, both studies demonstrate higher rates of major postoperative complications and early mortality when compared with our cohort, suggesting a comparatively favorable perioperative outcome profile in our patients. In a study by Yu et al., the median follow-up time was 55.8 months, the median DFS was 17.9 months (95% CI 0-36.2 months), and OS was 25.3 months (95% CI 14.8-35.8 months) [17]. The five-year DFS and OS were 28.5% and 30.8%. Compared to this study, our cohort shows slightly improved DFS and OS rates, possibly reflecting differences in patient selection, surgical techniques, or perioperative care. Long-term outcomes are broadly similar.

Although pelvic exenteration surgeries are associated with quite a high rate of morbidity and mortality, the procedure is still considered to be standard in the treatment of many of the gynecological malignancies. In earlier eras, this was considered a palliative procedure mainly for recurrent malignancies.

While the outcomes observed in our study appear favorable when compared with previously published series, these comparisons should be interpreted with caution. The retrospective nature, variations in case mix, including patient selection predominantly posterior exenteration, tumor characteristics, prior treatments, and institutional expertise, may significantly influence surgical and oncologic outcomes. Therefore, direct comparisons across studies may not be entirely appropriate. Larger, standardized studies are needed to allow meaningful comparative evaluation.

In this study, we evaluated the survival and morbidity in 60 patients who underwent pelvic exenteration for gynecological malignancy. Generally, pelvic exenteration surgery was associated with high intraoperative and postoperative morbidity. In our cohort, the most common intraoperative complication was hypotension with an average blood loss of 985 ml, which was managed by blood product transfusions and not associated with any postoperative complications. The most common postoperative complication observed was wound infection, which required wound re-suturing under local anesthesia. Anastomotic leak was observed in two (3.4%) patients. Most of the patients had ovarian cancer, and survival was not adversely affected by the more extensive exenteration procedure.

## Conclusions

In summary, pelvic exenteration is a definite option in indicated cases of advanced gynecological malignancies in the context of advanced surgical techniques, modern intensive care facilities, and surgical expertise. With respect to survival benefits, exenteration procedures are associated with an acceptable rate of major complications.
